# Factors contributing to uncontrolled hypertension in Ekurhuleni District, South Africa: the community health workers’ perspectives. A qualitative analysis

**DOI:** 10.1017/S1463423625100601

**Published:** 2025-11-13

**Authors:** Zaheerah Dawood, Kganetso Sekome

**Affiliations:** Department of Physiotherapy, School of Therapeutic Sciences, Faculty of Health Sciences, University of the Witwatersrand, Johannesburg, South Africa.

**Keywords:** Community health worker, high blood pressure, primary care, South Africa

## Abstract

**Background:**

In South Africa, community health workers (CHWs) provide home-based care and health promotion for patients with chronic conditions like hypertension. However, their views on patients’ poor blood pressure control remain unclear. Understanding CHWs’ perspectives could inform future community-level strategies for improving blood pressure management.

**Objectives:**

To explore CHWs’ experiences about factors contributing to uncontrolled hypertension among adults living in a South African District.

**Methods:**

A qualitative exploratory design, based on 22 face-to-face, semi-structured interviews with CHW. Data was transcribed and analysed manually using thematic analysis.

**Findings:**

Four themes emerged: (1) adequate knowledge about blood pressure and hypertension. However, lack of comprehension about the physiological concept of blood pressure and hypertension, (2) interventions used for hypertension control were contextualized and very simple, (3) contextual barriers to hypertension control included financial, personal, systemic, medication as well as cultural and traditional factors, (4) strategies to improve hypertension control included improved team work, awareness creation, holistic healthcare, improved access to clinic facilities, system-related improvements and patient initiatives.

**Conclusion:**

Community health workers (CHWs) demonstrated sufficient knowledge of hypertension, highlighting the need to strengthen and standardize their training, supervision, and support. Their patient advice was practical and context-specific. To address barriers and stigma around hypertension, CHWs should lead awareness campaigns and engage in community-based exercise and support groups, with help from rehabilitation teams. Collaborating with local police and community leaders to address violence, alcohol, and crime, along with initiatives like community gardens, multidisciplinary teamwork, and more frequent home visits, could further improve hypertension control.

## Introduction

Hypertension is a global public health concern with a worldwide prevalence of 22% among adults (Monakali *et al*., 2018). By the year 2025, 1.56 billion individuals worldwide will be suffering from uncontrolled hypertension (Kim and Oh, 2013). Uncontrolled hypertension is a driving factor for cardiovascular diseases (Mills *et al*., 2020). In South Africa, during the year 2000, hypertension was responsible for 47,000 deaths, but more recent data show that the prevalence has increased from 25% in 2000 to more than 40% in 2020 (Kohli-Lynch *et al*., 2022). Statistics show that 27.4% of South African men and 26.1% of South African women present with uncontrolled hypertension (Jongen *et al*., 2019).

Access to health care for the majority of the population in rural and peri-urban communities of South Africa is through the public health system offered via a primary health care service (Monakali *et al*., 2018). Regular blood pressure screening is performed by nurse practitioners and community health workers (CHW) at the primary health care clinic, and medication is prescribed when a hypertension diagnosis is made. The principle of primary health care emphasizes the need to provide health services where people live, work, and play (Walley *et al*., 2008). Research from other Sub-Saharan African countries has shown that there are positive health outcomes with the provision of community and home-based services, which are usually conducted by CHWs (Pillay and Barron, 2011). Community health workers have a contextual understanding of the communities they serve, which includes understanding the cultural beliefs and the languages spoken (Tsolekile *et al*., 2014; (Gaziano *et al*., 2014). Community health workers who are trained in hypertension management contribute to promoting awareness about the condition, which can have positive results on its control.

Research has been conducted to understand the hypertensive patients’ opinion regarding the increased levels and management of uncontrolled hypertension (Jongen *et al*., 2019). Hypertensive patients from a study conducted in rural South Africa recommended that contextual approaches to reducing high blood pressure should include starting a community garden, which enhances physical activity and promotes a good diet (Sekome *et al*., 2024). Only one known study has been conducted in South Africa, which investigated the beliefs, attitudes, and knowledge of CHWs regarding uncontrolled hypertension (Sengwana and Puoane, 2004). This study focused on understanding the CHWs’ perceptions regarding the epidemiology of uncontrolled hypertension from an urban district. This current study was able to establish the CHWs’ knowledge on measures used to control hypertension, explore barriers that contribute to uncontrolled hypertension, explore facilitators to improving the control of hypertension, and establish the CHWs’ perceptions on strategies used by hypertensive patients to improve the control of hypertension. No known studies in South Africa have investigated the perspective of CHWs regarding factors contributing to uncontrolled hypertension, especially for a semi-rural population. Due to the difference in geographical locations, there would be expected differences in cultural beliefs, differences in levels of education, diet, and access and quality of health care.

Our study aimed to explore CHWs’ perceptions on factors that contribute to uncontrolled hypertension in adults living in a semi-rural sub-district of South Africa. Gaining an understanding of the CHWs’ perceptions may contribute to future interventions focused on designing community interventions that are informed by the CHWs’ perspective, in addition to the patient perspective.

## Methods

### Research design

This was a qualitative exploratory study that used face-to-face, in-depth interviews guided by a semi-structured interview guide to understand the CHWs’ perspective (Nassaji, 2015).

### Population, sample, and sampling

The study was conducted at the Ekurhuleni District, Gauteng, South Africa. The population was sampled from four communities within the Ekurhuleni South sub-district. The researcher was employed as a community physiotherapist in the following district and worked closely with the CHWs in all four study sites. These communities were also chosen as the researcher had observed a high prevalence of stroke and cardiovascular diseases because of uncontrolled hypertension while working in the communities. Between the four study sites, there were a total of 38 employed CHWs; however, only 22 were interviewed due to data saturation. Data saturation was reached once the first author realized that no new ideas and themes were being discussed by the participants. To be included in the study, participants must have been involved in the management of patients with hypertension, have been working as a CHW at Ekurhuleni South sub-district for six months or longer, and have undergone the CHW basic training provided by the national Department of Health. Retired CHWs were excluded from the study.

### Data collection

A pilot study was conducted with three allied professionals (an audiologist, speech therapist, and occupational therapist) as well as two CHWs who were then excluded from the main study. Amendments were made to the interview guide where questions were amended so that they were more open-ended and so that they were more comprehensible. The interview guide started off by asking participants what they understood by blood pressure, then in-depth questions related to uncontrolled high blood pressure were introduced as the main areas of focus, according to the study objectives. These included barriers and facilitators to controlling high blood pressure, and strategies to improve the control of high blood pressure.

Revised face-to-face in-depth interviews were then conducted in English (interviews were conducted in English as the CHWs are trained in English) by the first author during March 2022 and April 2022 and were recorded with participants’ consent using a voice audio recorder device. Each interview lasted between 40-80 minutes. The first author contacted the CHWs’ managers at each of the study sites to explain the study. The managers gave the researcher a time, date, and a convenient place for the researcher to explain the study to CHWs. Those who were interested in the study signed the consent form, and a separate date and time for each participant was set to conduct the interview. Two consent forms were developed, one for participation and one for audio recording. The consent forms were handed out by the researcher to the CHWs at the respective clinics. Written consent was obtained from all eligible participants. Participants were assigned a letter of the alphabet to represent themselves to ensure anonymity. All interviews were conducted at the primary health care clinic facilities where the CHWs are employed in a private consultation room.

### Ethical considerations

Ethics was approved by the Human Research Ethics Committee of the University of the Witwatersrand (M210861). This research study was also registered with the South African National Health Research Database (GP202110028). Permission to make use of the four study sites was sought and granted by the head nurses at each study site.

### Data analysis

The audio-recorded files were transferred to the first author’s computer, and an external transcriber conducted transcription in Microsoft Word. The six phases of thematic analysis by Braun & Clark (2006) were used to analyse the data. Data were analysed manually by the authors, and findings were recorded in a Microsoft Word document. Manual analysis was deemed appropriate because it allows for a deeper dive into the content of the data. This method was also chosen because of the small data set. The authors began by reviewing transcripts and audio recordings to ensure accurate, verbatim transcription and to gain a deeper understanding of the data. Observations were noted, and data cleaning followed to correct any errors. In step two, the researcher developed a codebook through inductive coding, identifying and grouping similar comments from transcripts into codes. For instance, responses about managing hypertension (e.g., dietary changes, increased water intake, adequate sleep) were grouped under the theme ‘interventions used for the management of hypertension’. Codes were written on sticky notes and organized into themes, which were refined to ensure consistency and clarity. Themes were adjusted as needed for a better fit, then named and defined appropriately. Finally, conclusions were drawn from the themes and codes, with a supervisor reviewing and validating the coding process through inter-coder agreement.

### Findings

The participant’s demographics are described in Table 1. A total of 22 CHWs were interviewed; 20 (91%) were female and 2 (9%) were male. Females make up the majority of CHWs in the study population. The age ranged between 25–60 years (*m* = 40, SD = 8.59). Data analysis revealed four themes: (1) adequate knowledge about blood pressure and hypertension; (2) interventions used for hypertension control; (3) contextual barriers to hypertension control; (4) Strategies to improve hypertension control.

**Table 1.** Participants’ demographics

Reported gender	20 females; 2 males
Age	25–60 years; mean = 40 years
Number of years as a community health worker	4–13 years
Educational training received for community health work	Matric = 16; phase one CHW = 20; phase two CHW = 13; enrolled nursing = 4; health promotion = 2; home-based care = 1; primary health care = 1; pre-nursing = 1; disability management = 1; office administration

## Theme 1: Adequate knowledge about blood pressure and hypertension

During the interviews, participants referred to four sub-themes that explained their understanding of blood pressure and hypertension.

1. Definition of blood pressure: The CHWs had adequate knowledge about how to describe hypertension as seen by the quotation: ‘Blood pressure is a pressure of the blood against the arteries’ (CHW Q, phase I trained).

There were some CHWs who were unable to correctly define blood pressure and stated that it was high sugar levels in the body, while some CHWs defined hypertension by listing the causes, such as stress, poor dietary habits, and lack of exercise. A few were unable to define blood pressure at all. However, all CHWs were able to accurately provide the normal ranges for blood pressure.

1. Definition of uncontrolled high blood pressure: Most CHWs defined uncontrolled hypertension as exceeding the normal blood pressure values. Most of the definitions of uncontrolled hypertension by the participants included listing the causes and providing interventions for uncontrolled high blood pressure: ‘When you have uncontrolled high blood pressure it’s when the blood pressure cannot be controlled by the medication you are using due to some of the circumstances like lack of exercise, smoking, drinking and obesity’. (CHW W, phase II trained)

Very few CHWs were able to provide a more accurate definition of uncontrolled high blood pressure. A CHW mentioned:

*‘if they test you today they cannot diagnose you today, they tell you that you should come back and they will diagnose you, I am telling the patient that if they diagnose you, you are taking treatment then the high blood should be back to normal like from 120/60 [mmHg] or like if you are taking your pills correctly. But if it’s always 150/100 [mmHg] or 180/120 [mmHg], it means it’s uncontrollable high blood pressure’. (CHW K, phase I trained)*

1. Causes of uncontrolled high blood pressure: The Majority of the CHWs were able to provide a more accurate list of causes. Table 2 lists the perceived causes with quotations from the CHWs.
2. Complications of uncontrolled high blood pressure: Few CHWs listed complications that were related to Diabetes Mellitus, such as poor wound healing, amputation, and blindness; however, most CHWs were able to accurately list some of the complications related to uncontrolled high blood pressure, which included stroke, death, cardiac complications, and systemic complications. ‘It [hypertension] can lead you to stroke, and also lead you to renal failure, kidney failure, and heart failure’. (CHW M, phase I trained).

**Table 2.** Perceived causes of uncontrolled hypertension with participants’ quotations

Category	Perceived cause	Participant quote
Personal reasons for lack of adherence to medication and clinic follow-ups	Non-adherence to medication and clinic follow-up appointments Recreational substance abuse	*‘When the patient is not taking the medication as prescribed, not following the diet, and not coming to the clinic for their follow-ups’. (CHW W, phase II trained).* *‘Because many clients, or half of them who are taking alcohol mostly on weekends, are challenging because during the weekends they don’t comply with the treatment, so they relapse. We are supposed to educate them on how to take alcohol or to decrease to take alcohol’. (CHW A, phase I trained).*
Staff-related attitudes	Negative staff attitude Staff are not providing enough time for health education	‘Some of them they tell me they don’t want to come to the clinic because of the staff, that is why’. *(CHW P, phase II trained).* ‘For me, I feel like nowadays when you come in and go to the sisters (nurses) there’s this rush, they don’t sit one on one and give the health talks.’ *(CHW F, phase II trained).*
Drug-related causes	Dependence on anti-hypertensive drugs. Ineffectiveness of medication	‘I think a lot of the clients also they have this dependence on pharmaceutical treatment on tablets, if they don’t take the tablets, they don’t feel right’. *(CHW G, phase II trained).* ‘It’s either maybe the medication doesn’t work in your blood’. *(CHW T, phase II trained).*
Lifestyle-related causes	Poor dietary habits and lifestyle behaviour.	‘The other thing is that the people are smoking and drinking, that causes high blood pressure. You can drink, but control yourself so that your blood pressure can be fine’. *(CHW J, phase I trained).* ‘The causes are unhealthy diet, a lot of salt in food’ *(CHW F, phase II trained).*
Psychologically related issues	Stress	‘It’s caused by lots of stress, from stress it goes to depression, from depression it goes to high blood as far as I know, even when you take the alcohol, it makes your high blood pressure to be high. When you don’t solve your issues, you are in an abusive relationship, and you don’t want to get out, or you don’t have something to eat, and you have a child, you end up having stress because you don’t want to get out of that relationship. I think that is the thing that makes the high blood to go up.’ *(CHW I, phase I trained).*

## Theme 2: Interventions used for hypertension control

In their quest to assist hypertensive patients in controlling their blood pressure, the CHWs provided some valuable health education. This is seen via four sub-themes:

1. Improve dietary habits: CHWs stated that they educated and encouraged patients to improve their dietary habits by reducing the amount of spice, salt, oil, carbohydrates, caffeine, and sugar intake, and they encouraged patients to increase their fruit and vegetable intake. Furthermore, the CHWs educated patients on the importance of correct food preparation: ‘We teach them that they must not eat a lot of fat; they must take out the skin if it’s chicken, or beef they must take out the fats and that they must boil it, and they must not put a lot of salt’. (CHW J, phase I trained)
2. Improve mental health practices: Community health workers advise their patients to manage stress by talking to others regarding what stresses them out; they should try and avoid stressful situations, read a book, take a walk, and spend time talking to God. One of the CHWs stated the following regarding management of stress: ‘Oh and to avoid stress because old people are always crying to us, they say that we don’t listen, so we teach them that if you want to talk you can come to the clinic and talk to us so that you don’t have a lot of stress’. (CHW J, phase I trained)
3. Adherence to medication regimen: The Majority of the CHWs educate their patients regarding medication adherence. At one of the study sites, the CHWs all explained the 24-hour medication concept:

‘I tell them that another important thing is to take their medication the same time every day. Yes. And they must choose the time that is suitable for them. For instance, if they choose eight o’clock, it must be eight o’clock every day. If it’s bi-daily, then it must be eight [o’clock] in the morning and at night’. (CHW H, phase II trained)

1. Reduction of substance abuse: All CHWs reported a high prevalence of substance abuse amongst adults in all study sites. This leads to most hypertensive adults defaulting on their medication. In their interventions, CHWs emphasize the importance of reducing or stopping alcohol intake and tobacco smoking: ‘yoh! and the influence of alcohol is a lot here. That is why they don’t take the treatment seriously because they don’t take [medication] on Friday and Saturday as there’s a party, and you can’t take a Friday treatment on Sunday, that’s how they end up defaulting.’ (CHW I, phase I trained)

## Theme 3: Contextual barriers to hypertension control

Multiple contextual factors ranging from financial, social, system, cultural, and personal affected the patient’s ability to keep their blood pressure controlled.

1. **Financial issues:** In all study sites, CHWs report that most hypertensive patients are unable to afford the prescribed diet due to unemployment. Lack of affordability of healthier food items, therefore, leads to poor dietary habits: *‘What can I say? The environment is very poor. Like I said, lots of people are unemployed, another thing is that they can’t afford to buy the vegetables and stuff, they depend on social grants’. (CHW H, phase II trained)*
2. **Challenges in starting a garden:** The CHWs, however, educate hypertensive adults to initiate their vegetable gardens, but the adults complained that they do not have gardening space and have insufficient funds to start a garden of their own: ‘Yeah, and we also educate them on how to do their garden as well. Most of them ask us where they are going to get the money to buy the seeds’. (CHW D, phase I trained)
3. **Personal and stigma-related issues:** Denial of condition and fear of diagnosis disclosure, especially to family members, were two of the most common issues that CHWs raised. A CHW referred to denial of the diagnosis through patients who report that: ‘some patients deny and say: how could I have high blood because I am still young, I am not taking my treatment because I am in denial, that I don’t have that’. (CHW K phase I trained).
4. **Poor peer influence:** There appears to be sharing of anti-hypertensive medication amongst community members due to a lack of knowledge about the danger. Friends also encourage each other to consume alcohol and to make use of certain ‘concoctions’ to assist with controlling high blood pressure.

‘Sometimes other people don’t give good advice; they are bad influencers. So, the friend will go to the patient’s house and say ‘hey come on, let’s go to what-what’, the patient will say ‘I still have to take my medication’, the friend will say ‘no don’t worry that one you will take them when you come back let’s go do what-what’. And then if they go somewhere like sports bars, the patient will also start drinking [alcohol]’ (CHW U, phase II trained).

1. **Domestic violence:** High levels of domestic violence were reported by CHWs in all study sites, which often leads to patients defaulting on medication. Due to alcohol abuse, the male partner becomes physically abusive when intoxicated and will physically abuse the hypertensive female. Due to this additional stress, which the hypertensive female underwent, she often forgets to take her medication. Another scenario is where the male partner is the sole breadwinner, and his income is insufficient to support the household. This leads to no food being put on the table every evening which causes the male partner to abuse the female thus leading to an increase in stress and the hypertensive female defaulting on her medication:

‘if only the government can be a little bit stricter on the people who are selling alcohol and drugs, then I think the levels can go down even the level of violence can go down because even in the houses there’s domestic violence because there the men who drink alcohol they come back and beat their wives and children’. (CHW Q, phase I trained)

1. **Clinic and system issues:** The CHWs alluded that patients complain of long queues at the local clinics when attending follow-up appointments. Medication shortage was also another system issue identified. One of the CHWs said: ‘Mostly people default due to the service at the clinic. They feel like they don’t want to come back due to Nurse A or B’s attitude and due to the long waiting time’. (CHW C, phase II trained).
2. **Traditional and alternative medicine use:** In some study sites, CHWs noted that patients still made use of traditional healers and alternative medicine. In one of the study sites, a CHW is quoted, ‘This belief that they [community members] have and they go to their traditional healers and they give them this medication that they drink, now they mix or they don’t even drink their medication from the clinic, they drink whatever they get from the traditional healers.’ (CHW R, phase II trained)
3. **Patient expectations from health services:** Community health workers stated that patients expect to get something other than education from CHWs; they expect things like food vouchers.
4. **Changes in medication packaging:** Community health workers reported that sometimes the medication packaging changes without the patients being made aware. Patients then default on medication as they do not know that it is the same medication, just a different packaging: ‘I think because the medication is the same, but the boxes are changed. Because most of the patients, for example, Metformin, they know normally it was in a white box but now it comes in a green box. And then they will not drink because they want that white box.’ (CHW D, phase I trained)

## Theme 4: Strategies to improve hypertension control

The strategies recommended by CHWs to improve the control of hypertension varied from community development, improving the wellness of staff and enforcing teamwork, creating awareness about hypertension, providing a holistic care approach through collaborating with other stakeholders, increasing clinic operating hours, and initiatives that must be taken by the government.

1. **Social and community development:** Community health workers recommended that the government should provide communities with seeds, ploughing equipment, and an allocated community space to enable them to start up their own gardening projects.
2. **Increased community health workforce and improved infrastructure:** Community health workers suggested that the government should employ more nurses, as there is a shortage of nurses in all study sites. This will decrease patients’ waiting time and allow nurses more time for health education. Another suggestion given was that the government should employ more CHWs, as they are also short-staffed and the population they serve is large.
3. **Inter and intra collaboration:** Social media should be more involved in health education by teaching about hypertension and stroke. Effective communication between all health professionals was encouraged. The CHWs stated that if all health professionals communicated well together, common goals would be achieved.
4. **Patient-centred approach:** Community health workers alluded to the importance of health professionals assessing the socioeconomic circumstances of the patient prior to management. The importance of understanding the patient’s contextual challenges and goals was also highlighted; this way, the CHWs can provide a more focused approach.
5. **Staff wellness, teamwork, and leadership:** The importance of improving teamwork and communication channels amongst CHWs and clinic staff members was highlighted. Community health workers stated that they are seen as a separate entity, and they do not receive the support that they deserve from clinic staff members. They expressed that clinic staff members did not understand their role as CHWs and they underestimate the value and achievements they bring.
6. **Awareness creation:** Community health workers recommended that greater awareness within the communities should be done; their recommendations included campaigns for hypertension, exercise classes for hypertensive patients, improving family education, creating support groups for hypertensive patients, and health awareness at community meetings. High blood pressure screening could also be conducted for all community members regardless of blood pressure status.
7. **Traditional healers and church collaborations:** The study participants recommended collaboration with traditional healers. In their recommendations, they stated that traditional healers should receive education on referring patients to medical facilities. Collaboration with the churches was also suggested. By collaborating with the churches, CHWs can work with the priest at the church to encourage the patients to attend their follow-ups and to come to the medical facilities to seek medical assistance. Certain church members believe that once the priest prays for you and you drink the holy water, you will be cured, and you do not have to take your medication any longer.
8. **Hypertension clinic initiative:** The CHWs suggested that a blood pressure clinic should be initiated at each local clinic. This will improve awareness, treatment, and control of hypertension. The hypertension clinic will ensure constant medication supply and can also assist in reducing the stigma associated with hypertension due to increased visibility and health education opportunities.
9. **Community self-screening for blood pressure: The** Government should investigate the feasibility of providing patients with blood pressure machines. This will empower them as they will be able to monitor their blood pressure closer to home. The provision of blood pressure machines can be centralized in a common community structure, such as a shopping centre or church.
10. Upskilling CHW training: The CHWs also requested that the government provide continuous training to all CHWs so that they are up to date with recent advances in hypertension. They also suggested that the government should conduct annual evaluations to assess how effective CHWs are with patients.

## Discussion

Our study revealed that CHWs had adequate knowledge about hypertension and its associated causes, which is contradictory to previous research in South Africa by (Tsolekile *et al*., 2018) who reported that CHWs in their study possessed poor knowledge regarding the risk factors and complications of hypertension. Another earlier study in South Africa also reported that CHWs have insufficient knowledge regarding the causes of uncontrolled hypertension (Sengwana and Puoane, 2004). These findings may point to an improvement in the training of CHWs regarding hypertension. The education of hypertension in South Africa has been gaining momentum as it has been identified in the quadruple burden of disease (Bradshaw *et al*., 2019).

Financial difficulties have been reported by previous research as one of the main factors contributing to poor dietary habits for hypertensive patients (Wexler *et al*., 2009, Shrestha *et al*., 2018). This finding was also revealed in our study, as CHWs report that their patients lack the finances to purchase healthier food options as recommended by the health educator. A recent study in rural South Africa by Sekome *et al*., (2024) also reported similar findings. The study by Sekome and colleagues recommended that advice given to hypertensive patients should not focus on purchasing a new diet, but rather on adjusting the existing diet to lower salt and calorie intake. CHWs in our study reported stigma around being hypertensive and taking chronic medication, which led to their patients defaulting on medication and not making the necessary lifestyle adjustments. This has been reported elsewhere where younger hypertensive patients perceived hypertension to be a disease of the elderly and refused to take chronic medication when young (Gebrezgi *et al*., 2017).

In another study conducted in Tanzania, it was reported that there was a stigma around using lifelong medication as it was linked to HIV treatment (Kisigo *et al*., 2022). There seems to be a need for community-based awareness campaigns to reduce the stigma around hypertension and chronic diseases. There is poor support from family and community members in our study population, as reported by our study participants. This led to hypertensive patients engaging in alcohol consumption, consulting traditional medicine, and sharing medication due to not wanting to be seen going to the primary health care clinic for chronic medication. We could not find any study regarding these concepts in South Africa; however, a study conducted in Northern Ghana reported that community members in their population are supportive of behavioural change approaches to control hypertension (Nyaaba *et al*., 2018). Another study in Tanzania also reported that family members are key supporters of clinic attendance and medication adherence for adults with hypertension (Kisigo *et al*., 2022).

A unique finding in our study population, which contributes to poor medication adherence, especially for female adults, is gender-based violence due to alcohol abuse and gangsterism from the male partner. The physical and emotional abuse also leads to increased stress levels, thus causing high blood pressure. A study in the Kyrgyzstan Republic by (Abba *et al*., 2022) highlights that there is an association between gender-based violence and hypertension.

Clinic staff attitudes and clinic understaffing were some of the system-related factors contributing to poor education about hypertension control and long waiting times in our study. These findings are not unique to our study, as they have also been reported elsewhere (Shrestha *et al*., 2018); however, other settings have reported contradictory findings where African American adult patients stated that they received all the required hypertension education, and when they attended their follow-up clinic appointments (Rimando, 2015). The education they received was due to a structured health education service offered by mid-level workers at the clinic, a concept that South African clinics can adopt. Other strategies which can be adopted in the South African context are making use of Tertiary learning students in the health sector, such as physiotherapy, nursing, medicine, pharmacy, and nutrition. This strategy is already employed in some South African institutions at the primary health care level (Cobbing, 2021, Sekome *et al*., 2023).

CHWs in our study expressed that the communities they serve were populous and they were unable to cover the entire community; therefore, some patients were left undiagnosed, untreated, and follow-ups could not be conducted. The CHWs suggested that self-guided blood pressure screening in the community should be implemented. This could take the form of installing high blood pressure self-screening machines in common local areas such as schools, shopping centres, pharmacies, and churches. This type of approach has been tested in Australia and has shown that community-based health checks may identify people with high blood pressure and could provide an option for self-monitoring. Broader implementation is thus needed to increase the reach in rural areas and among the elderly population (O’Hagan *et al*., 2024).

When asked to propose strategies to assist in hypertension control, CHWs in our study mostly suggested the provision of vegetable seeds, ploughing equipment, and space to start a community or family gardening project. This was also seen as a means to promote physical activity. There is an opportunity to explore this approach, as no known study in South Africa has tested this intervention before. A systematic review by Hume *et al*. (2022) reported that community gardening results in higher fruit and vegetable intake; however, the association between community gardening and physical activity and hypertension was mixed, with one study by Hawkins *et al*. (2011) reporting no changes in body mass index (BMI), physical activity, and hypertension between community gardeners and people in their usual activity pursuits. Studies looking at testing/implementing community gardening projects should look at enhancing the intensity of physical activity participation.

CHWs in our study recognized the need to strengthen their training and continuous development. They see value in the continuous monitoring & evaluation of their services so that gaps and needs are identified that will allow the government to improve their services. This is not a unique finding, as Tsolekile *et al*. (2014) have previously reported that continuous training and development of CHWs is fundamental in strengthening the services CHWs offer. Another aspect to consider in strengthening CHW services is to foster collaboration between CHWs and other healthcare providers. The team-based care approach is discussed by Carey *et al*. (2018), who explain that the patient is the centre of the approach, surrounded by the multidisciplinary team (MDT) of nurses, pharmacists, social workers, CHWs, and allied professionals. The MDT team works to complement the primary physician by providing support and sharing the responsibility of the hypertensive patient. This allows more time for the physician to assist patients with more complex conditions. Odedosu *et al*. (2012) stated that making use of a team-based care approach resulted in the greatest improvement in the management of uncontrolled high blood pressure (HBP).

To strengthen hypertension control at the community level, CHWs in our study suggested that household visits to hypertensive patients should happen more frequently. A suggestion was made to conduct these visits at least once or twice weekly. A study by Ribeiro *et al*. (2011) supports this finding by stating that personalized, frequent home visits have shown an improvement in dietary change, and a reduction in salt and sugar intakes not only for the patients but for the family members as well. They also reported reductions in BMI, weight, glucose, systolic blood pressure, and waist circumference.

## Conclusion

Community health worker programme needs to be strengthened by improving their training through continuous development, monitoring, and supporting of their services. There is an urgent need for interventions to improve awareness of hypertension to the wider community, and not just the hypertensive patient. This will go a long way in reducing stigma associated with a chronic disease diagnosis and can enhance social support and medication adherence. More focus will have to be given towards running exercise and support groups in all the communities of this study population. Community health workers will have to work with multidisciplinary disciplines, such as the local police forum and community leaders, to assist with social and socioeconomic barriers in all the communities. Community-based interventions for hypertension control must also focus their efforts on providing low to no-cost strategies for the patients, such as self-guided monitoring of blood pressure and promoting community-based garden projects. Increasing the frequency of household visits to hypertensive patients can go a long way in improving the control of hypertension.

## Limitations

While this study is relevant globally, generalization of findings can be done to contexts like the Ekurhuleni sub-district in Gauteng, South Africa. It should be noted that the generalization of findings in qualitative research is not the primary focus. Even though the CHWs stated that their preferred language was English, more detailed responses could have been obtained if the interviews were conducted in the CHWs’ home language.

## Data Availability

Upon reasonable request, data regarding this study can be made available from the corresponding author.
